# The United States Pharmacopœia; Revision of 1880

**Published:** 1883-04

**Authors:** C. E. Clacius

**Affiliations:** 322 W. Madison St.


### ARTICLE III.

THE UNITED STATES PHARMACOPŒIA. Revision of 1880. By C. E. CLACIUS, PH.G., M.D.

This long-expected volume has been received, perused and criticised with all the curiosity and interest due to its importance. Although no law makes it obligatory upon any physician to prescribe the remedies formulated therein, or upon any pharmacist to keep them, as it is merely an agreement between the regular medical and pharmaceutical professions, that the latter shall have on hand certain remedies, with which the former will treat their patients, it is a matter of great importance, that this agreement be strictly observed, to secure all over the country a uniformity of those remedies in effect, taste and appearance, which is essential for the final result, viz: the healing of the sick, and for the material benefit of the profession.

This latest revision of the pharmacopœia is particularly interesting, more so than its predecessors; not so much for the numerous additions of new remedies or the omission of obsolete ones; not so much for the improvement of the classical Latin, or for the restoration of the masculine gender to some names which for the last twenty years have erroneously passed for feminines, but for the abolition of the ancestral system of weights and measures, and the substitution therefor of *parts by weight*, in decimal proportions. This is a decidedly progressive step, and it is only to be regretted that the step is a little too short. The old octesimal system, with its Troy ounces of 480 grains and its avoirdupois ounces of 437 grains; with its pounds of twelve and of sixteen ounces; with its imperial gallons of 160 and apothecaries' gallons of 128 ounces, has existed too long, and given much annoyance to all who had to use them. Therefore the new arrangement, however defective it may be, is welcomed as the initiatory step toward a great improvement: a uniformity of weights and measures in every business, the introduction of the metric system in this country. Although continental Europe has introduced this system long ago, and although it has been accepted in scientific literature all over the world, the United States have, since they gave up the shillings and sixpences for dimes and half dimes, done nothing toward a uniformity among themselves and with other nations in the measure of values. The people of this country still use pennyweights, various kinds of ounces, bushels and Fahrenheit's thermometer, in all of whose favor nothing can be said except that the fathers have used them.

The new pharmacopœia has now adopted a decimal system, at least in the proportional strength of the preparations. That strength was formerly in drachms to ounces, and ounces to pints; now it is in proportions of 1, 5, 10, 15, 20, etc., in 100 parts. Thereby of course the strength of every single remedy is changed, of some in a higher, of others in a lesser degree, and while, in the large majority, the difference is hardly worth noticing, it is in some of the preparations of considerable importance.

Like every innovation and improvement, so brings this, beside its advantages, considerable temporary trouble and annoyance. The physicians, in making their prescriptions, have to accommodate their calculations of doses to the new order of things, and it cannot be denied that it will be quite a task for the memory of the practitioners to become familiar with the new strength of the remedies which they are in the habit of using. The pharmacopœia has on pages 454 and 455 a table, which comparatively shows the difference in the revisions of 1870 and 1880 of such remedies where either the remedy or the difference, or both, were of sufficient importance to deserve special attention. That table is given below, and to facilitate the desirable acquaintance with the names, those remedies now stronger have been separated from those which are now weaker than in the former issue.

For the pharmacist, the trouble caused by the innovation is of a different character. His memory is not taxed much, because he has the book with the formulæ always on hand for information, but in this transitory period from the old to the new formulæ, he is occasionally called to account by the consumers of the medicines for a difference in taste and appearance of remedies, which, however slight it may be, is noticed by some sharp tongue and eye, and to avoid being suspected of having made mistakes, he has to keep on hand for the repetition of old prescriptions, the preparations of the old issue, beside the new ones. That state of affairs, however, will be only transitory, the new rule will soon reign supreme, and the old one be forgotten.

Besides the difference in the strength of remedies in the new issue, caused by the adoption of decimal proportions, another factor has to be taken in account by the physician, when he contemplates the dose for his patient, viz: the specific gravity of the fluids. The decimal proportion is calculated for the weighed fluid, not for the measured; but in dispensing, the fluid is measured, not weighed. The measured fluid ounce of 480 minims of an ordinary tincture, made with diluted alcohol, weighs only 450 grains, and the weighed ounce of 480 grains of the same fluid measures 510 minims; thus we have in 510 minims the decimal proportion of 480 grains (in a tincture of 10 in 100, 48 grains, not the decimal proportion of 510), because the alcohol weighs so much less than water. In the tinctures made with stronger alcohol, the difference is still greater; there the measured ounce weighs only about 420 grains, and the substance belonging to 480 grains is in 570 minims. Further, tinctures, made of an equal quantity of substance, and with the same menstruum, vary in specific gravity, because some drugs contain more extractive matter than others which enters into the liquid and increases the gravity. On the other sides are the heavier fluids. A fluid ounce of syrup weighs 660, but 480 grains have the proportional strength of an ounce. Take for instance, syrup of ipecac. The new formula is: fluid extr. ipecac, 5 parts; simple syrup, 95 parts (of course by weight); the calculation would be, that the strength of that syrup is one in twenty, and that a teaspoonful contains 3 grains of the root, but it is not so; the fluid ounce of 480 minims has the strength of 660 grains, which means 1 in 16, or 4 grains to the teaspoonful. Thus the physician's calculations are somewhat marred by the specific gravity, and this is caused by the directions to use parts *by weight.*

The committee on revision state in the preface to the Pharmacopœia, that when they directed in the formulæ the use of parts by weight, they did so partly by the instructions of the convention which appointed them, partly with the intention to adjust the proportions of the new formulæ, so that the new preparations would not differ materially from the old ones. Is not this strange? The strength of every single remedy is changed for the sake of having decimal proportions, and then again are the decimal proportions sacrificed in order to keep nearer the old strength. The remedies are directed to be made by weight, while they are prescribed, dispensed and taken by measure; the trouble of calculating the doses is increased for the physician by bringing into question the specific gravity, merely to make the difference a little smaller, while a difference remains. If the committee of revision had made the progressive step a little farther, and had left out the words by weight; if they had adopted in all formulæ grammes and cubic centimeters, as they have done in the manufacture of fluid extracts, the physicians in calculating doses would have found a little more difference in the strength, with which, however, they would have become familiar as soon as with the smaller, but then the formulæ would have been more permanent, while, as it is, the committee of the next revision in all probability will have to cut another inch off the dog's tail, by converting the fluid weight into measure again.

Among the remedies which have been greatly affected by the new rule, opium and its preparations deserve to be mentioned.

Not only have the liquid preparations changed in proportional strength, powdered opium itself, of which they are made, has been raised in its standard morphine value. While the former issue directed that no opium should be used containing less than 10 per cent. morphine, the new one says that opium shall not contain less than 12 nor more than 16 per cent. morphine. Although the chances of morphine in opium are thus somewhat contracted, there are still 4 per cent. left in which it can vary, which shows that opium, though one of our most valuable remedies, is a variable drug. That this new ruling in regard to the morphine strength in opium will cause any particular change or difference is very doubtful, for the reason that opium of the standard strength of the old revision, that is of 10 per cent. morphine value is such a poor quality in the market that it never has been used by pharmacists who are able to judge and analyze opium, and conscientious enough to buy drugs of best quality. Those men, it may be asserted, have always used an opium of such quality as the new revision requires, consequently there will be no change. Such pharmacists, however, who have never known nor cared about the quality of opium they have used, will hardly change their tactics now.

This change in the standard strength of opium, together with the increased proportion of the drug to the tincture under the new decimal system, has given rise to various widely different opinions about the comparative strength of the former tincture and the present. The two extremes appear to be on one side: the committee of revision, who, in the mentioned differential table gives the strength of the former 9 in 100, of the present 10 in 100, difference 10 per cent. On the other side, Dr. E. R. Squibb, who, in the Ephemeris of November, 1882, states, that the present tincture is 50 per cent. stronger than the former. Let us see. The former pharmacopœa directed to make 1 quart $=$ 32 fl. ounces of tincture of $2\frac{1}{2}$ ounces of opium, that makes $37\frac{1}{2}$ grains in one fluid ounce of 480 minims, or 1 grain $12=\frac{1}{2}$, or 8 in 100. Where the committee get their 9 in 100 is a mystery. The new tincture has 10 grains in 100, if we overlook the small difference caused by the specific gravity, by which there are 48 grains in 510 minims instead of in 480. According to these figures 4 drops of the new tincture would be equal to 5 of the former, and physicians may feel pretty safe in such calculation. Doctor Squibb, in his estimate, gave the difference in the morphine strength too much consideration, and, for reasons stated above, is theoretically more right than practically.

The deodorized tincture—the wine—which formerly contained 1 grain in 8, the vinegar, which had 1 grain in 6, also the new preparation, tincture of ipecac and opium, or tincture of Dover's powder, have all now the same opium strength of 1 in 10, as Dover's powder always has had. Dover's powder is made with sugar of milk instead of sulphate potassa, which may occasionally be the cause of inquiry from people, who notice a difference in taste.

The arsenical solutions, liquor potassæ arsenitis and liquor acidi arseniosi have been advanced in strength from 4 grains in 1 ounce (480 minims) to 4 grains in 400, or 1 in 100; 5 drops of the present equal to 6 drops of the former.

The diluted mineral acids have suffered a change; in order to bring them to the decimal proportion, diluted hydrochloric acid had to be made stronger, diluted nitric acid had to be reduced, as is shown in the table; diluted nitro-hydrochloric acid, which the table does not mention, has been reduced from 25 in 100 to 19 in 100.

Extract of aconite, which was formerly made of the leaves, is now made of the root. Extract and tincture of conium are now made of the fruit or seed instead of the leaves, because of the superior strength of the substituted drugs. But we find nowhere an answer to the question: How much stronger are the new proportions than the former? How can physicians who have been prescribing extract of conium in full doses, know how much of the new will have the same effect as the old; if the new is much stronger, harm may be done if the pharmacist uses it, when the physician expects to have the old. What can the pharmacist do in such cases? If he has used up all the old extract, of course he prepares the new; perhaps he keeps both kinds, but how shall he know which to give? In this transitory period it would be advisable for physicians, when prescribing any of the remedies which have changed much in strength, to signify which they intend to have. Very desirable it would have been, if the committee of revision, who undoubtedly had experimented and found the difference in the strength before they directed the change, had said something about the differential doses.

Among the additions the eye is met first with the new remedies: Abstracts, dry sacharated extracts of twice the strength of the crude drug and fluid extract, and about half the strength of the solid extract. They were adopted "because a demand had arisen for them," the committee says. Writer has some curiosity to know whence that demand came. The pharmacies keep already of those remedies, the crude drug, tincture, fluid extract, solid extract, and generally, also, the resinoid; what is to be done with the abstract? It is not to be presumed that physicians will commence to give their patients extracts in powder form at an age when medicines, much less objectionable to the taste, have to be covered with sugar or gelatine or given in sweet elixirs to be tolerated. It does not appear desirable to have the 75 per cent. of sugar of milk in ointments or suppositories, or in pills. In the latter it would increase the size unnecessarily, as so much inert matter, while the solid extract in combination with quinine or other powders acts not only as medicine, but also as vehicle to form the mass. An argument has been made in favor of the abstracts, viz: that the solid extraets in the market are to some extent made of the drugs, after the latter's exhaustion by tinctures or fluid extracts, consequently did not represent the medicinal value of the drugs. The reply to that would be, that no pharmacist should buy his extracts, he ought to make them, then he knows they are good, provided he makes them right, and whosoever does not make his own extracts will not make the abstracts, and the chances of being imposed upon by inferior preparations are the same. The only advantage apparent in the use of abstracts is a greater facility for the pharmacist to weigh 5 or 10 grains; that, however, seems hardly of sufficient importance to introduce a new series of medicines so nearly alike those already on hand.

A valuable addition is the compound powder of glycyrrhiza, which for a number of years past has gradually worked into the favor of practitioners. It originally was a sort of arcanum, composed and used by a Doctor Kurella, who used it more for throat and pectoral troubles than as a laxative; later it was adopted by various European pharmacopœias, which, up to this date, give as a synonym, "Pulvis pectoralis Kurellæ." The new pharmacopœa in adjustment of the decimal proportions has increased the quantity of senna by 2 per cent., which seems very proper, as it is used here mostly for its laxative virtue.

The numerous additions are found in a table on pages 436 to 441. Many valuable remedies can be noticed there; some quite new; others not new, but now found worthy of a place in the pharmacopœia. One formula is in the book for—it is difficult to say what; it is not a remedy, not prescribed by physicians, and the majority of pharmacists would never use it, the spiritus myrciæ or artificial bay rum; it stands there with a solitary impropriety, and suggestion has been made that it either should have company by formulæ for artificial cognac and for communion wine, or should itself be banished.

Many of the profession were disappointed not to find in the new issue a number of formulæ for elixirs, a demand having arisen for them for a long time, the neglect of which severely injures the interests of both professions. There are a number of combinations which have proved valuable and which are constantly prescribed, but if the pharmacopœia gives no formulæ, by which the pharmacists can uniformly make them, their manufacture will be continued outside of the profession by a few parties, who will place them in the market like patent medicines.

The manufacturers introduce them to the public through the physicians, and in a short time the public obtains them everywhere without the assistance of the medical profession. There are many people now who buy their quinine, quinia, iron, strychnine pills and others at the wholesale houses. Elixirs likewise—and they may soon buy them in any grocery, if the indifference to the mutual interest continues. Some standard formulæ for combinations, mostly used, of elixirs, for a standard emulsion of cod liver oil, with which the physicians could combine anything they wish, would cause much improvement; the formulæ are known to most of the intelligent pharmacists, and all that is necessary to be done, is, to establish them as a standard for uniformity.

Since the pharmacopœia has not given any of these formulæ, it is to be hoped that the dispensatories or commentaries, which generally follow its new issues, will supply the want, and that the professions interested will, in such cases, recognize them as standard.

PRINCIPAL CHANGES AND DIFFERENCES IN THE STRENGTH OF PREPARATIONS OF THE NEW AND OLD PHARMACOPŒIAS.

| STRONGER IN THE NEW ISSUE. | 1870. | 1880. | WEAKER IN THE NEW ISSUE. | 1870. | 1880. |
|---|---|---|---|---|---|
| Acidum aceticum | 35. | 36. | Acetum lobeliæ | 13. | 10. |
| " " dilutum | 4.5 | 6. | " opii | 16.3 | 10. |
| " hydrochloric, dilut. | 7.8 | 10. | " sanguinariæ | 13. | 10. |
| " phosphoric, " | 9.8 | 10. | " scillæ | 13. | 10. |
| Alcohol dilutum | 39.3 | 45.5 | Acidum nitricum dilutum | 11.6 | 10. |
| Confectiio Sennæ | 8.32 | 10. | " sulphuric, dilut | 12.1 | 10. |
| Extractum aconiti | Leaf. | Root. | " sulphurosum | 6.4 | 3.5 |
| " conii | Leaf. | Seed. | Ferri et quiniæ citras (quinine) | 16. | 12. |
| Liquor acidi arseniosi | 0.87 | 1. | Liquor potassæ | 5.8 | 5. |
| " ferri chloridi | 35. | 39. | Spiritus camphoræ | 14. | 10. |
| " potassæ arsenitis | 0.87 | 1. | Tinctura aconiti | 47.6 | 40. |
| Opium pulvis | 10. | 12.16 | " aloes et myrrhæ | 12 | 10. |
| Spiritus anisi | 6.8 | 10. | " arnicæ | 23. | 20. |
| " cinnamomi | 8. | 10. | " colombæ | 15. | 10 |
| " juniperi | 2. | 3. | " cannabis indicæ | 36. | 20. |
| " lavandulæ | 2. | 3. | " cinchonæ | 25. | 20. |
| " menthæ piper. | 6.4 | 10. | " cubebæ | 15. | 10. |
| " menthæ viridis | 6.4 | 10. | " guajaci | 23. | 20. |
| " myristicæ | 2. | 3. | " " ammoniata | 23. | 20. |
| Tinctura aloes | 3.3 | 10. | " nucis vomicæ | 3.5 | 2. |
| " asæfœtidæ | 16. | 20. | " serpentariæ | 15. | 10. |
| " cantharidum | 3.5 | 5. | " veratri viridis | 55. | 50. |
| " capsici | 3.5 | 5. | " zingiberis | 31.8 | 20. |
| " catechu composita | 7. | 12. | Unguentum acidi carbolici | 12. | 10. |
| " conii | Leaf. | Seed. | " belladonna | 12. | 10. |
| " gauac | 15. | 20. | " gallæ | 12. | 10. |
| " humuli | 17.5 | 20. | Vinum opii | 13. | 10. |
| " myrrhæ | 12. | 20. | " rhei | 14. | 10. |
| " opii | 9. | 10. | | | |
| " opii deodorata | 9. | 10. | | | |
| " quassiæ | 6. | 10. | | | |
| " rhei | 10. | 12. | | | |
| " valerianæ | 15. | 20. | | | |
| " valerian. ammon. | 15. | 20. | | | |
| Unguentum acid. tannic. | 6. | 10. | | | |
| " hydrargyr. ammoniat. | 8. | 10. | | | |
| " hydrargyr. oxyd. flav. | 8. | 10. | | | |
| " zinci oxydati | 16. | 20. | | | |
| Vinum ergotæ | 12.5 | 15. | | | |

322 W. Madison St.

